# Diagnostic and therapeutic approaches in deep neck infections: an analysis of 74 consecutive patients

**DOI:** 10.1016/j.bjorl.2020.07.002

**Published:** 2020-08-13

**Authors:** Kemal Koray Bal, Murat Unal, Nuran Delialioglu, Ragip Onur Oztornaci, Onur Ismi, Yusuf Vayisoglu

**Affiliations:** a University of Health Sciences Adana City Hospital, Department of Otorhinolaryngology & Head and Neck Surgery, Adana, Turkey; b University of Mersin, Faculty of Medicine, Department of Otorhinolaryngology, Mersin, Turkey; c University of Mersin, Faculty of Medicine, Department of Microbiology, Mersin, Turkey; d University of Mersin, Faculty of Medicine, Department of Biostatistics and Medical Informatics, Mersin, Turkey

**Keywords:** Deep neck infections, Surgery, Anaerobic bacteria, Antibiotics, Mortality

## Abstract

**Introductıon:**

Deep neck infections are a group of diseases with serious complications and mortality, which can occur as a result of common diseases in the community and which have surgical and medical treatment options.

**Objectives:**

Patients ages, genders, complaints, physical examination findings, hospitalization complaints, history of antibiotic use before the application, additional diseases, radiological tests and analysis of examinations, type of treatment method, antibiotic agents selected in treatment, bacterial culture results, duration of hospitalization, complications, mortality rates were systematically recorded. In the study, anaerobic bacterial factors, which are difficult to produce in routine, were produced by considering special transport conditions and culture media.

**Methods:**

A total of 74 patients who were hospitalized in the Department of Otorhinolaryngology, University of Mersin, between 01.07.2016 and 01.07.2017 for deep neck infection were evaluated prospectively. The study included 37 female and 37 male patients. The ages of the patients ranged from 1 to 69 and the mean age was 31 years.

**Results:**

According to the analysis of the obtained data, there was a statistically significant relationship between the patients with additional diseases and the treatment modalities of the patients (*p* = 0.017). The surgical treatment rate was increased in this group of patients. In patients with a history of antibiotic use, it was found that patients in the pediatric group were in hospital longer in terms of length of stay compared to adults (*p* = 0.036). In adult patients who underwent surgery, the absorptive long axis was found to be longer in mm than in patients receiving isolated medical treatment (*p* = 0.008).

**Conclusions:**

Deep neck infections is a disease group that seriously concerns public health, with significant mortality and morbidity. Ensuring airway safety of patients should be the first intervention. Abscesses located lateral to the tonsil capsule may not drain adequately without concomitant tonsillectomy.

## Introduction

Deep neck infections (DNI) were first described by Galen in the second century.^1^ DNI occurs in potential cavities and fascial recesses in the head and neck region.^2^ These infections lead to soft tissue cellulitis and phlegmon, progressing frequently to life-threatening abscesses. Although the frequency of deep neck infections decreases after the use of antibiotics, there are still problems in diagnosis and treatment. Delay in diagnosis and treatment can lead to serious life- threatening complications, as they can spread from potential anatomical cavities.^3^ Upper respiratory tract and salivary gland infections, odontogenic infections, trauma, foreign body and surgical manipulations are the most common sources of infection for DNI.^4^, ^5^ In the absence of broad spectrum antibiotics, DNI was most commonly caused by tonsillopharyngitis and more frequently developed within the parapharyngeal area. The prevalence of tonsillopharyngitis- induced DNI decreased due to widespread use of antibiotics in the early period. Currently, DNI is often developed in the submandibular area due to odontogenic and salivary gland infections. In children, peritonsillar involvement due to acute tonsillitis is the most common cause. Submandibular area involvement with odontogenic origin is the second most common cause.^6^, ^7^, ^8^, ^9^ Recent studies have shown that neck drug injection in intravenous drug addicts plays an important role in the etiology of DNI. However, the source of infection cannot be determined in 20%−50% of the patients.^7^, ^8^ Diagnosis has become more complex after frequent use of antibiotics. Recent studies show that about half of patients use antibiotics without admission. Symptoms such as edema, fluctuation, swelling decrease and systemic findings are suppressed in these patients. As a result, delay in diagnosis and the incidence of complications that can be prevented increase.^8^ Anamnesis, clinical symptoms and findings, additional laboratory and radiological examinations are the factors useful in diagnosis.^10^, ^11^

Symptoms and signs vary according to disease progression and site of involvement. The most common are pain, fever and swelling.^12^, ^13^ In addition, edema, redness, heat increase, dysphagia, odynophagia, trismus, dyspnea, laryngeal edema, stridor, toothache, dehydration, dysphonia, restlessness, crying, agitation, loss of appetite, neck mass, torticollis, neck mobility, dental anomaly, oropharyngeal anomaly and laryngeal anomaly may accompany the typical signs and symptoms. The duration of these symptoms can range from 12 h to 28 days.^11^, ^14^, ^15^ The number of pathogens in DNI is quite high. *Streptococcus species* and *Staphylococcus aureus* are often the causative pathogens, although most of the patients have aerobic and anaerobic bacteria, which are often found together. In most studies, aerobic bacteria are cultured more frequently. This may be due to the fact that anaerobic bacteria are more difficult to produce and to identify in cultures.^16^, ^17^, ^18^, ^19^, ^20^, ^21^ The main principles of treatment include: airway safety, medical antibiotic treatment and surgical drainage. Treatment and supportive treatment of the source of infections should not be forgotten. Hospitalization of patients should always be considered. Another point that should be considered is that atypical signs and symptoms may occur in immunocompromised and diabetic patients. Complications should also be more strongly considered in these patients.^8^, ^22^, ^23^ Despite effective diagnostic methods, broad spectrum antibiotics and surgical procedures, deep neck infection can be fatal.^8^, ^11^, ^24^

Our aim was to detect anaerobic bacteria and compare efficacy of surgical-medical treatment in different age groups.

## Methods

This study was carried out with prospective evaluation of 74 patients who were hospitalized at the Department of Otorhinolaryngology, University of Mersin between 01.07.2016 and 01.07.2017 for deep neck infection. The study was approved by University of Mersin Clinical Research Ethics Committee dated 26/11/2015 and numbered 2015/360. Patients who were hospitalized for deep neck infection and underwent surgical and medical treatment were included in the study. Medical files, computed tomography scans and intraoperative photo images were recorded for each patient.

### Diagnosis, surgery, and medical treatment

Patients were categorized as pediatric (0 − 18 years) or adult (18-). In this study, patient’s age, sex, time from the onset of their complaints to admission to the hospital, history of antibiotic use before admission to hospital, radiological imaging studies, value of the long axis of abscesses in millimeter (mm), physical examination findings, symptoms, additional diseases, radiological and surgical placement of abscess. locations, localization of infections, treatment method, intraoperative findings, bacteriological culture results, pathologic results of specimens taken from surgical procedure, surgical operation method, penicillin allergy, empirical antibiotic treatment, need for change in antibiotic treatment, presence of complications, duration of hospitalization, total antibiotic usage time, need for intensive care and death, tracheotomy need, drugs, alcohol and smoking were recorded. Abscess locations were divided into 7 regions and defined. These regions; Level − 5 (atypical), parapharyngeal, peritonsillary, submandibular, SCM medial (Level 2 – 3 − 4), parotid area and submental area. Complaints and symptoms of the patients were grouped under 7 titles including pain, swelling, trismus, dysphagia, fever, neck movement limitation and dyspnea. Physical examination findings were classified as parotid swelling, bulging of the upper tonsillar pole, distorted uvula, submandibular swelling, swelling in the medial of SCM, swelling in the posterior cervical triangle, mandibular swelling (on mandibular corpus), and submental swelling. Surgical and medical therapy (intravenous antibiotics) were used in 61 of 74 patients treated for deep neck infection. Isolated medical therapy (i–v antibiotics) was preferred in 13 patients. All patients underwent surgery under general anesthesia. Endotracheal intubation was preferred in patients undergoing general anesthesia. One or more of the surgical methods, such as abscess drainage, tracheotomy, neck exploration, unilateral tonsillectomy and sialoendoscopy, were preferred in patients according to the location of their infections. All 74 patients were hospitalized and received iv antibiotic therapy. Among the selected antibiotics, ampicillin + sulbactam, clindamycin, meropenem, teicoplanin, ciprofloxacin, ertapenem, sultamicillin, cefazolin, anidulafungin, linezolid, piperacillin + tazobactam were employed. Antibiotic drug selection was decided by consulting the pediatric infectious diseases and adult infectious diseases departments according to the patient's age, additional diseases, and whether he had previously received antibiotic treatment. Antibiotic treatment was reviewed according to the results of the cultures and the response of the patients.

### Microbiology

Culture was obtained from 60 patients in order to produce aerobic and anaerobic agents in microbiology laboratory.

### Tools and equipment

Devices used and used in microbiology laboratory

1 Oven at 37 °C (Memmert, UK)
2 Biosafety cabinet (Thermo Scientific, USA)
3 Autoclave (Hirayama HV-L Seies 50 L)
4 Anaerobic jar (Genbox, Biomerieux, France)

### Used kits and chemicals

1 Anaerob media provider (Genbox anaer, BioMerieux, France)
2 Anaerobic indicator (BioMerieux, France)
3 Anaerob bacteria identification rapid ID-32A kit (BioMerieux, France)
4 Vancomycin A disk (5 micrograms) − BOX 1 (50 DISC) (Bioanalyse, Turkey)
5 Colistin A (10 micrograms) − one box (disk 50)) (Bioanalyse, Turkey)
6 Kanamycin A disc (1000 micrograms) − 1 box (50 discs) (Bioanalyse, Turkey)
7 SPS Disc −50 discs BBL taxo, (Becton-Dickinson, USA)

### Used media

#### Anaerobic basal agar (CM0972-OXOID, UK)

For 1 liter of distilled water, 46 g of medium was added, sterilized by autoclaving at 121 °C for 15 min, after cooling to 50°−55 °C and sheep blood was added to sterile defibrin and poured into sterile petroleum.

#### Thioglycolated medium (Himedia, India)

29.75 grams of powder medium was added to 1 liter of distilled water, sterilized by autoclaving at 121 °C for 15 min with stirring, and 5 mL was dispensed into sterile glass tubes.

#### Sheep blood agar (RTA, Turkey)

Media poured into ready to use petroleum

#### Chocolate agar

40 g of blood agar base (Himedia, India) powder medium is added for 1 liter of distilled water and it is sterilized by autoclave at 121 °C for 15 min after mixing. The medium was cooled to 70 °C and sheep blood was added to sterile defibrin and poured into sterile petroleum.

### Collection and transport of samples

Deep neck abscess samples taken during the operation were delivered to the laboratory in sterile tube and thioglycolated medium.

### Cultivation of samples

Aerobic and anaerobic planting of the samples were performed without delay. Sheep blood agar, chocolate agar and anaerobic blood agar medium were planted. Sheep blood agar medium was incubated under aerobic conditions in the oven, chocolate agar desiccator (wax jar) and anaerobic blood agar were incubated under anaerobic conditions. In order to provide anaerobic media, jars of anaerobic media provider (Genbox anaerob, BioMerieux, France) and anaerobic indicator were placed, and the lid was closed, and the oven was removed. Two samples were prepared and Gram stained. All media were incubated at 35°−37 °C for 48 h.

### Evaluation of cultures and identification of isolates

At the end of incubation, aerotolerance test was performed by making passages on chocolate agar medium from each colony grown on anaerobic media. Bacteria that grew in anaerobic medium and did not grow on chocolate agar in 5%−10% CO_2_ were evaluated as anaerobic bacteria. The colony structure was examined for identification and the hemolysis properties of the colonies were evaluated for pigment formation. Gram stained preparations and morphology of each colony were examined. Bacterial suspension was prepared for identification of anaerobic bacteria and spread over the whole area of the anaerobic blood agar medium by swab. Subsequently vancomycin to distinguish Gram-negative bacilli (5 μg; Bioanalyse, Turkey), colistin (10 μg; Bioanalyse, Turkey), kanamycin (1000 μg; Bioanalyse, Turkey) discs were placed. To distinguish *Peptostreptococcus anaerobius* from other anaerobic Gram-positive cocci, sodium polyethanol sulphonate (SPS; BBL taxo, Becton-Dickinson, USA) disc was used. In addition, anaerobic bacteria identification was performed at species level using rapid ID-32A Strips (BioMerieux, France) Rapid ID-32A is a system consisting of 29 enzymatic tests that identify anaerobic bacteria within 4 h. 4 MacFarland turbidity bacterial suspension is prepared, the suspension is distributed to 55 microliters of strips until 32 wells are added to the urea well, the lid is closed and incubated under aerobic conditions for 4 h. NIT1 + NIT2, IND well JAMES and PAL well and serA well were added to the NIT well and 10 min after the FB reagent was dropped, reactions were evaluated according to the evaluation table and the identification number was obtained. These numbers were obtained by entering the apiwebTM identification software. Species name was obtained. Gram positive cocci were evaluated by Gram staining, catalase, DNAase, esculin hydrolysis, bacitracin and optokin sensitivities.

### Statistics

Statistical analysis of the data was performed with SPSS 21.0 trial version statistical package program. Descriptive statistics were given for numbers and percentages for categorical variables and Chi-Square test was used to show the relationship between two categorical variables. Kolmogorov-Smirnov and Shapiro-Wilks tests were taken into consideration for the control of the normal distribution of continuous variables. If the difference between the groups was determined as a result of the Chi-Square evaluations, if the number of categories was more than two, the control of the difference was evaluated by two − rate *t* test and Bonferroni correction p-values ​​were used. Median, first and third quartile values ​​were given as descriptive statistics for categorical type variables and the nonparametric alternative Mann-Whitney *U* test was used in order to compare the mean of two groups in cases where appropriate conditions were not provided for independent samples *t* test. Kruskal Wallis statistic, a non-parametric alternative to one-way ANOVA, was used in cases where the data did not conform to the normal distribution in terms of a continuous variable with more than two groups. In order to determine which group was the difference, post hoc test was applied based on corrected p-values. Statistical significance level (p) was taken as 0.05 for all analyzes.

## Results

The study was conducted on 74 patients 37 female (50%) and 37 males (50%). The ages of the patients ranged from 1 to 69 years. The mean age was 31.34. While the mean age for women was 30.57, the mean age for men was 32.97. The study included 13 pediatric (0–18 years) and 61 adults (18–69 years) patients. Seven (53.8%) of the pediatric patients were female and 6 (46.2%) were male. Thirty (49.2%) of the adult patients were female and 31 (50.8%) were male.

The time period from the onset of complaints to the hospital admissions ranged from 0 days to 47 days. The average admission period was 7.32 days. Nineteen patients had comorbidities (25.7%) and 55 did not have comorbidities (74.3%).Additional diseases included hypothyroidism, hypertension (HT), celiac disease, pregnancy, coronary artery disease (CAD), rheumatoid arthritis (RA) + immunosuppressant use, chronic obstructive pulmonary disease (COPD), cirrhosis, diabetes mellitus (DM), chronic kidney disease (CKD), amputated lower extremity, penicillin allergy, esophageal squamous cell cancer (esophageal SCC) and ankylosing spondylitis.

There was no statistically significant difference between the patients with comorbidities and those without comorbidities in terms of hospitalization time (*p* = 0.137). There was a statistically significant relationship between the presence of comorbidities and treatment modalities. (*p* = 0.017). All patients with additional disease underwent surgical treatment.

Fifty-six patients (75.6%) had a history of antibiotic use before admission. Antibiotics were prescribed by a family doctor or by secondary or tertiary health care facilities. Some patients had a history of self-antibiotic use without examination. Eighteen patients (24.4%) had no history of antibiotic use before admission. Of the 13 pediatric patients, 11 (84.6%) had a history of antibiotic use, and 2 (15.4) did not. 45 (73.8%) of the adult patients had a history of antibiotic use, while 16 (26.2%) had no history of antibiotic use. There was no statistically significant difference between the pediatric and adult groups in terms of hospitalization duration in patients without a history of antibiotic use (*p* =  0.327). There was a statistically significant difference between the pediatric and adult groups in terms of hospitalization time in patients with a history of antibiotic use; it was concluded that pediatric patients were hospitalized for a longer period (*p* =  0.036).

Twenty-five (33.8%) of the patients had a history of smoking and 7 (9.5%) had a history of both smoking and alcohol use. No patients had drug use or addiction. 42 patients (56.8%) were not using alcohol, smoking or drugs. There was no statistically significant difference between smokers and nonsmokers in terms of hospitalization time (*p* =  0.051). There was no statistically significant difference between isolated smoking and treatment modality (*p* =  1.00).

Complaints and symptoms of the patients were recorded as pain, swelling, trismus, dysphagia, fever, neck movement limitation and dyspnea. Pain in 73 patients (98.6%), swelling in 38 patients (51.4%), trismus in 16 patients (21.6%), dysphagia in 7 patients (9.5%), fever in 28 patients (37.8%), neck movement limitation in one patient (1.4%) and dyspnea in 2 patients (2.7%) were present. The most common complaint was pain.

The most common physical examination was bulging in the upper pole of the tonsil and displacement of the uvula (41.9%) (Table 1).

**Table 1:** Physical examination findings.

Physical examination	Frequency	Percentages
Groups		
Parotid swelling	4	5.4
Submandibular swelling	20	27.0
The medial of SCM (Sternocleidomastoid Muscle) swelling	7	9.5
The posterior cervical triangle swelling	2	2.7
Submental swelling	4	5.4
Mandibular swelling (mandibular corpus)	1	1.4
Bulging in the upper pole of the tonsil and pushing in the uvula	31	41.9
Swelling in Submandibular + SCM medial + Posterior cervical triangle	1	1.4
Submandibular + Parotid swelling	1	1.4
Bulging in the upper pole of the tonsil and pushing in the uvula + Submandibular swelling	1	1.4
Submandibular + SCM medial swelling	2	2.7

Sixty-six patients (89.2%) underwent computed tomography (CT) and 5 patients (6.8%) underwent ultrasonography (USG). Three patients (4.1%) did not undergo CT. CT radiologic examination was the most common (85.2%). Three patients who were inappropriate for radiological examination were adults and their abscesses were peritonsillar and submandibular. The long axis of abscess was measured. The abscess long axis was found to be 5 mm minimum and 76 mm maximum. In pediatric patients, the minimum was 5 mm and the maximum was 45 mm. There was no statistically significant relationship between the abscess long axis value in mm and the age of the patients (pediatric − adult) (*p* =  0.229). There was a statistically significant relationship between the value of the abscess long axis in mm and the treatment modality of all patients (*p* = 0.005) (Table 2). There was a statistically significant relationship between the value of the abscess long axis in mm and treatment modality in adult patients (*p* = 0.008). In pediatric patients, there was no statistically significant relationship between the value of the long axis of abscess in mm and the type of treatment (*p* = 0.318). While the value of the long axis of abscess in mm in surgical patients was larger than the group of isolated medical treatment, there was no such difference in the pediatric age group.

**Table 2:** Aerobic bacteria.

Aerobic bacteria	Frequency	Percentages
Groups		
Methicillin Resistant Staphylococcus Aureus (MRSA)	3	6.5
Klebsiella pneumoniae	2	4.3
*Streptococcus spp*. (alpha hemolytic streptococcus)	16	34.7
Escherichia coli	1	2.1
Methicillin Sensitive Coagulase Negative Staphylococcus (MSCNS)	11	23.9
Non A Non B Group Beta Hemolytic Streptococcus	2	4.3
Brucella Melitensis	1	2.1
Methicillin Sensitive Staphylococcus Aureus (MSSA)	3	6.5
Streptococcus pyogenes	1	2.1
Methicillin Resistant Coagulase Negative Staphylococcus (MRCNS)	3	6.5
Streptococcus mitis/oralis (alpha hemolytic streptococcus)	1	2.1
Streptococcus pneumoniae	1	2.1
Mycobacterium tuberculosis	1	2.1

When the abscess locations of the patients were examined, it was evident that 30 patients (40.5%) had peritonsillar abscess. The most common abscess area was the peritonsillar region. In pediatric patients, the most common abscess was seen in the medial SCM (30.8%), whereas peritonsillar abscess was the most common in adult patients (44.3%) (Table 3).

**Table 3:** Abscess locations.

Abscess locations		Frequency	Percentages
Groups	Level 5	2	2.7
	Parapharyngeal	10	13.5
	Peritonsilllar	30	40.5
	Submandibular	10	13.5
	Medial of SCM	9	12.2
	Parotid Space	2	2.7
	Submental	4	5.4
	Peritonsillar + Parapharyngeal	2	2.7
	Submandibular + Medial of SCM + Level 5	1	1.4
	Submandibular + Parapharyngeal	1	1.4
	Submandibular + Medial of SCM	3	4.1

There was no statistically significant difference between patient age and the location of the abscess (*p* =  0.063). The most common etiologic factor was tonsillopharyngitis (51.4%). Tonsil was the most common source of infection in 8 pediatric patients (61.5%) and 30 adult patients (49.2%) (Table 4).

**Table 4:** Etiology of infections.

Etiology of infections		Frequency	Percentages
Groups	Folliculitis (nape skin)	1	1.4
	Parotitis	2	2.7
	Infected branchial cyst	2	2.7
	Lymphadenitis	6	8.1
	Tonsillopharyngitis	38	51.4
	Odontogenic Infection	20	27.0
	Nasopharyngitis	2	2.7
	Submandibular gland sialoadenitis	2	2.7
	Tonsillopharyngitis + Odontogenic infection	1	1.4
	Total	74	100.0

There was a statistically significant difference between the locations of infections and patients' ages (*p* =  0.006). The nasopharynx, which is the source of infection, is a statistically significantly more frequent source of infection in pediatric age group (*p* =  0.0018).

All patients received iv antibiotics. In 61 patients (82.4%), surgical treatment was combined with medical treatment. Isolated medical treatment was selected in 13 patients (17.6%). Eleven pediatric patients (84.6%) underwent surgery and 2 pediatric patients (15.4%) underwent medical treatment. Fifty adult patients (82%) underwent surgery and 11 adult patients (18%) underwent medical treatment. There was no significant relationship between the treatment modality and the age of the patients (pediatric – adult) (*p* =  1.00). There was no statistically significant difference between the location of the abscess and the treatment modality (*p* =  0.343).

Abscess drainage in 24 patients (32.4%), neck exploration in 24 patients (32.4%), abscess drainage plus unilateral tonsillectomy in 10 patients (13.5%), abscess drainage plus tracheotomy in 1 patient (1.4%), neck exploration plus sialoendoscopy in 1 patient (1.4%) and neck exploration plus tracheotomy in 1 patient (1.4%) was performed. The most common surgical procedures were abscess drainage and neck exploration (32.4%). Two patients underwent tracheotomy due to additional problems on the days following treatment. None of the patients underwent tracheotomy due to difficult intubation or complications during the first surgical procedure.

Of 61 patients who underwent surgical treatment, 55 (90%, 2) had intraoperative abscess and 6 (9.8%) had cellulitis and/or necrotic lymph nodes. Intraoperative abscess, cellulitis and/or necrotic lymph nodes in patients had no statistically significant relationship with radiological abscess in one area and abscess in multiple areas (*p* = 0.136). There was a statistically significant relationship between the presence of abscess, cellulitis and/or necrotic lymph nodes in the patients and the presence of bacteriological culture (*p* =  0.01) Bacteriological culture was more likely to occur in patients with intraoperative abscesses. There was no statistically significant relationship between the age (pediatric/adult) and the presence of abscess, cellulitis, and/or necrotic lymph nodes intraoperatively (*p* =  0.294).

The most commonly used empirical antibiotic treatment was ampicillin sulbactam plus clindamycin combination. (66.2%) Empirical antibiotic therapy was changed in 7 patients (9.4%) due to lack of improvement in clinical status and culture results. Linezolid, piperacillin plus tazobactam, meropenem and teicoplanin were used in these patients according to culture results (Table 5).

**Table 5:** Empirical antibiotic therapy.

Empirical antibiotic		Frequency	Percentages
Groups	Clindamycin	6	8.1
	Ampicillin + Sulbaktam	12	16.2
	Ertapenem	1	1.4
	Sultamicillin	3	4.1
	Cefazolin	1	1.4
	Meropenem + Teicoplanin	1	1.4
	Clindamycin + Ciprofloxacin + Anidulafungin	1	1.4
	Ampicillin + Sulbaktam + Clindamycin	49	66.2

Patients were given iv antibiotic therapy during their hospitalization. Routine antibiotic treatment was continued to each patient for 10 days after discharge. The mean duration of antibiotic use was 17.99 days. Complications were observed in 3 patients (4.1%). Upper airway obstruction was seen in 1 patient (1.4%), premature birth in 1 patient (1.4%), mediastinitis plus pleural effusion and septic shock and upper airway obstruction in 1 patient (1.4%). One of the patients in the study (1.4%) died. Two patients (2.7%) needed observation in the intensive care unit. Two patients (2.7%) required tracheotomy during their hospitalization. Complications, requiring intensive care, tracheotomy and death were all in adult patients. Material was obtained for pathological examination from 36 patients (48.6%). Malignancy was detected in 1 (2.7%) of the patients with pathology (cervical esophagus squamosus cell carcinoma). This patient was pregnant, and her abscess was medial to the SCM. Preterm birth occurred in this patient.

Culture was obtained from 60 patients (81.1%). Bacterial growth was detected in 51 (85%) patients. Nine (90%) of 10 pediatric patients who had culture were found to have growth. Forty-two (84%) of the 50 adult patients who were cultured had positive growth. The most frequent bacterial growth was from the peritonsillar area. (39.2%) Isolated aerobic bacteria were isolated in 29 (48.3%), aerobic plus anaerobic bacteria (mixed, polymicrobial) in 12 (20%), and isolated anaerobic bacteria in 10 (16.7%). No bacterial growth was observed in 9 patients (15%). Most often isolated aerobic bacteria were cultured. (48.3%) (Table 6).

**Table 6:** Bacterial growth status in patients.

Bacterial growth status in patients		Frequency	Percentages
Groups	Aerobic + Anaerobic (mixed, polymicrobial)	12	20.0
	Aerobic	29	48.3
	Anaerobic	10	16.7
	No bacterial growth	9	15.0

The peritonsillar region was the most common site of abscess in patients with culture and bacterial growth; twenty patients (39.8%). There was single bacterial growth in 33 (64.7%) patients and more than one bacterial growth in 18 (35.3%) patients. Seven of 9 (77.8%) patients with no bacterial growth had a history of antibiotic use before admission. Of the 41 patients with aerobic bacterial growth, 32 (78%) were adult patients and 9 (22%) were pediatric. *Streptococci* were detected in 20 (48.7%) and staphylococci in 19 (46.3%) of 41 patients with aerobic bacterial growth. Of the 41 patients with aerobic bacterial growth, microbiological bacterial culture revealed 46 times aerobic bacterial growth. The most common bacteria (34.7%) were *Streptococcus spp.* (alpha hemolytic streptococcus) (Table 2).

Twenty-two patients with anaerobic bacterial growth identified 27 anaerobic bacteria as a result of microbiological bacterial culture. The most common bacteria were produced 5 times (18.5%); *Peptostreptococcus spp.*, *Prevotella spp.*, Prevotella oralis (Table 7).

**Table 7:** Anaerobic bacteria.

Anaerobic Bacteria		Frequency	Percentages
Groups	Peptostreptococcus spp.	5	18.5
	Prevotella spp.	5	18.5
	Prevotella oralis	5	18.5
	Veillonella spp.	1	3.7
	Porphyromonas spp.	1	3.7
	Campylobacter uratolytic (Bacteroides uratolytic)	2	7.4
	*Bacteroides spp*.	1	3.7
	Prevotella intermedia	4	14.8
	Fusobacterium nucleatum	1	3.7
	Prevotella melaninogenica	1	3.7
	Porphyromonas endotalis	1	3.7

There was no statistically significant relationship between aerobic, anaerobic, mixed bacterial growth and the sites where the infections originated (*p* = 0.954). There was no statistically significant relationship between aerobic, anaerobic, mixed bacterial growth and abscess sites (*p* = 0.604). There was no statistically significant relationship between aerobic, anaerobic, mixed bacterial growth and age of patients (pediatric – adult) (*p* =  0.081). There was no statistically significant relationship between the history of antibiotic use before the admission to the hospital and the growth of aerobes, anaerobes and mixed bacteria (*p* =  0.753).

The hospitalization time of the patients ranged from 2 to 22 days, with an average of 8.28 days. There was a statistically significant difference between pediatric and adult patient groups in terms of hospitalization time (*p* =  0.017). Adult patients had less hospitalization time than pediatric patients. There was no statistically significant relationship between the duration of hospitalization and whether patients had comorbidities (*p*  = 0.137). There was no statistically significant relationship between hospitalization time and abscess in single or multiple areas. (*p* =  0.746). Patients were operated on a mean of 1.80 days after admission (minimum 1, maximum 13 day).

Intraoperative images and laboratory images are shown below (Figs. 1–8).

**Figure 1:**
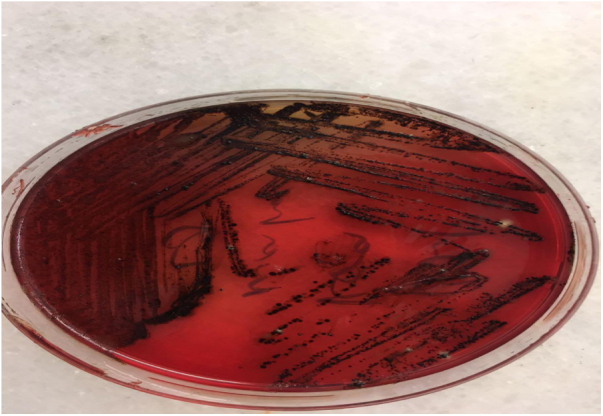
*Prevotella spp*. reproduction of colonies.

**Figure 2:**
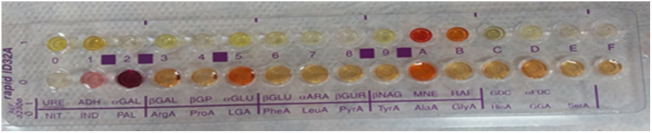
Prevotella oralis rapid ıd 32 a identification strip.

**Figure 3:**
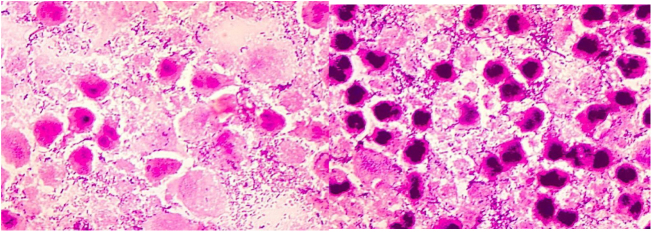
Prevotell intermedia microscopic image.

**Figure 4:**
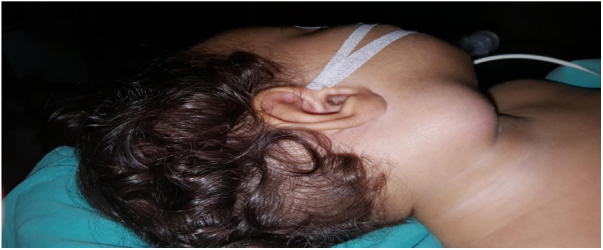
Preoperative Image of right submandibular abscess.

**Figure 5:**
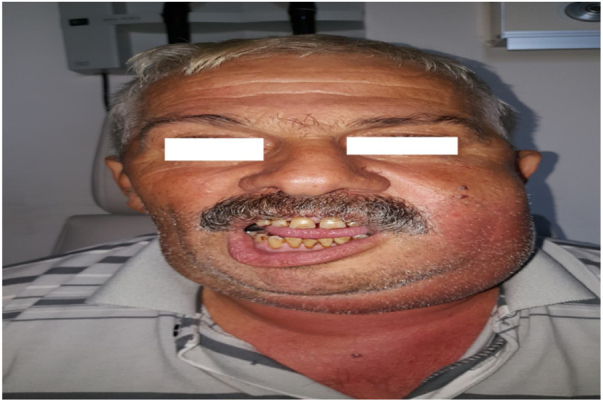
Preoperative image of a patient with trismus.

**Figure 6:**
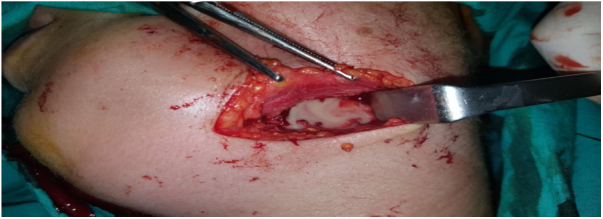
Drainage of left submandibular abscess.

**Figure 7:**
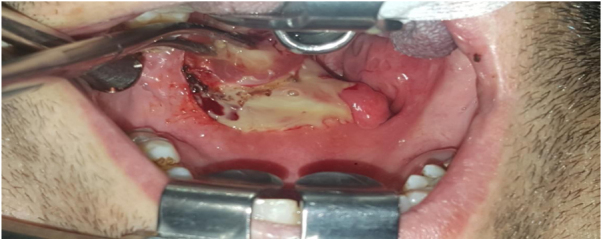
Drainage of the left peritonsillary abscess.

**Figure 8:**
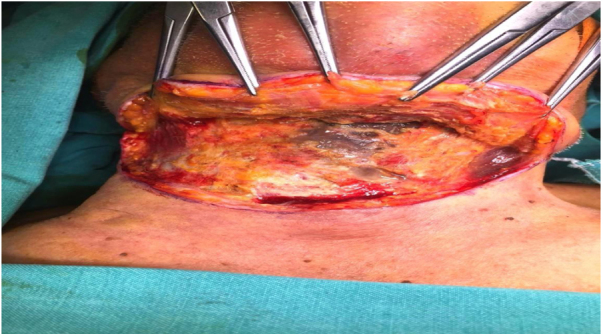
Necrotizing fasciitis eschar tissues after abscess in sternocleidomastoid medially.

## Dıscussıon

Cordesmeyer reported 63, Shimizu 123, Pascual 330, Adovica 263, and Ban 97 patients.^2^, ^25^, ^26^, ^27^, ^28^ There were 74 patients in our study, 37 males and 37 females. The ages of the patients ranged from 1 to 69 and the mean age was 31.34 years. There were 13 pediatric and 61 adult patients.

In Adovica's study, the most common etiologic cause was odontogenic infection; in 139 (70.6%) patients.^27^ In a study of 330 patients by Pascual, the etiology could be detected in 296 patients, 277 (83.9%) were tonsillopharyngeal infections.^26^ In the study of Shimizu, lymphadenitis (73%) was the most common cause in children, upper respiratory tract infections (45%) and odontogenic infections (29%) in adults.^25^ In the study of Çetin in 12 pediatric patients, tonsils and upper respiratory tract infections were found to be the most common etiologic cause in 11 (91.6%) patients.^29^ Crespo, in a study of 65 patients, odontogenic infection was the most common cause (43%) and the second most common cause was tonsillopharyngeal infection with 40%.^30^ Mayor studied 31 patients; in 21 (67.7%) patients, the etiology could not be detected^7^ In Celakovsky's study, with 634 patients, in 80% the most common cause was odontogenic infections.^31^ In the study of 76 patients of Kataria, in 26 (34.2%) patients the cause was odontogenic and in 21 (27.6%) patients, tonsillopharyngeal infection.^32^ In our study, 38 (51.4%) patients had tonsillopharyngeal infections, the most common cause. The second etiologic cause was odontogenic infections with 20 (27%) patients.

In the Adovica study of 263 patients, in 95 (36.2%) patients, the most common site of abscess was the submandibular region.^27^ In Pascual's study with 330 patients, in 215 patients (65.2%) the most common site of abscess was the peritonsillar region.^26^ In Kauffmann's study with 63 patients, the parapharyngeal region was the most common site with 24 (38.1%) patients, followed by peritonsillar region with 19 (30.2%) patients.^33^ In Crespo's study of 65 patients, the most common site of abscess was the parapharyngeal region with 65% followed by submandibular region with 60%. Crespo excluded patients with peritonsillar region abscess and patients without preoperative CT.^30^ Varghese found Ludwig's angina in 14 (33.3%) of patients from a total of 42. ^34^ In our study, the most common site of abscess was the peritonsillar region with 30 (40.5%) patients. The submandibular region (13.5%) and the parapharyngeal region (13.5%) were the second most common site of abscesses with 10 patients. In 4 of 13 pediatric patients in our study, the location of the abscess was medial to SCM. (most commonly, 30.8%) In the pediatric group, the second most common site of abscess was the peritonsillar region. In 27 of 61 adult patients in our study, the location of abscess was the peritonsillar region. (most commonly, 44.3%) In the adult group, the second most common site of abscess was the submandibular region(10 patients, 16.4%).

The most common clinical finding in Pascual's study was odynophagia with 98.2%, followed by trismus with 55.5%.^26^ The most common symptom in Kauffmann's study was sore throat in 96.8%, followed by neck swelling in 92%.^33^ Sichel's study of isolated parapharyngeal area infections in 7 patients (6 pediatric, 1 adult) showed fever and neck swelling in all patients.^35^ In Mayor's study of 31 patients with parapharyngeal and retropharyngeal infections, the most common symptom was found to be odynophagia 83.8%.^7^ The most common symptom in our study was pain with 98.6%. The most common physical examination findings were displacement of the uvula and bulging of the tonsil upper pole (41.9%).

In the Adovica study, 194 patients (58.8%) underwent CT examination.^27^ In the Kauffmann and Crespo studies, all patients (100%) underwent CT.^30^, ^33^ Sichel reported 7 pediatric patients with isolated parapharyngeal area abscess; CT scan was performed in each patient. In some patients, CT and magnetic resonance imaging (MRI) were repeated. CT was performed 11 times and MRI was performed twice.^35^ In the Mayor study, all of the 31 patients (100%) required CT, with routine CT repeated every week.^7^ In the Freling study, the positive predictive value of CT in deep neck infections was found to be 82%. It is said that the presence of air bubbles indicates a higher rate of abscess formation, with or without collection.^36^ In the Holt study, 22 patients underwent CT and 6 (27.2%) patients had CT and operative abscesses; false negativity and positivity rate was 0%.^37^ In our study, CT was performed in 66 (85.2%) patients and USG was performed in 5 (6.8%) patients. Radiological examinations were not performed in 3 (4.1%) patients and all of them were adult (2 patients peritonsillar abscess, 1 patient submandibular abscess). In our study, the mean long axis of abscess was 27.62 mm.The mean long axis in pediatric patients was 29.23 mm (20 mm − 45 mm), and in adult patients was 27.26 mm (5 mm − 76 mm).

In the Kataria study, DM was detected in 8 (10.5%) patients, i-v drug use in 4 (5.2%) patients, and chronic kidney disease in 2 (2.6%) patients. While 16 (21%) patients had a history of smoking, 28 (36.8%) patients chewed tobacco.^32^ Huang reported the DM rate as 30.3%.^38^ Crespo identified DM in 4 (6.1%) patients and AIDS in 4 (6.1%) patients.^30^ In the Kauffmann study, the most common cardiopulmonary (43%) comorbidities were detected, followed by DM with 19%.^33^ In our study, 19 patients (25.7%) had additional disease. Pregnancy status was evaluated in additional disease category. The most common comorbidities were pregnancy status (5.2%), DM (5.2%) and penicillin allergy (5.2%). In our study, 25 (33.8%) patients had a history of isolated smoking and 7 (9.5%) patients had both smoking and alcohol use. No i-v drug use was present in any of our patients.

In the Kauffmann study, 62% of patients were administered amoxicillin/clavulanate potassium, 14.3% of patients 2nd and 3rd generation cephalosporin and 4.7% of patients clindamycin received empirical initial antibiotic treatment. In a series of 63 patients, these antibiotics and their combination were successful in 33 patients, while the spectrum was expanded in 21 patients. Nine patients developed total resistance to all of these antibiotics which resulted in prolonged hospital stay. All patients underwent surgery under general anesthesia. Seven (11.1%) patients required revision surgery. Multiple abscesses were seen in 6 (85.7%) patients who underwent revision surgery.^33^ The rate of surgery in the literature ranges from 60% to 100%.^39^, ^40^ Çetin administered i–v antibiotics to 12 pediatric patients with parapharyngeal and retropharyngeal area infections. Patients were not treated surgically.^29^ Pascual preferred antibiotics with a combination of beta lactam plus beta lactamase inhibitors in 304 (92.1%) patients. Other preferred antibiotics are aminoglycoside, quinolone, clindamycin, metronidazole. Antibiotic resistance was seen in 3 (0.9%) patients and treatment was changed, with carbapenems preferred. Pascual gave antibiotic treatment to the patients for an average of 10.92 days (minimum: 4 days, maximum : 35 days). In this study, 245 (74.2%) patients underwent surgery, of which 196 (80%) were transoral and 36 (14.7%) were performed as an external cervical approach. These two methods were combined in 4 (1.6%) patients, as 9 (3.7%) patients with peritonsillar abscess required tonsillectomy during drainage. Sixteen (6.5%) patients who underwent surgery required revision surgery.^26^ In the Adovica study, 246 (93.5%) patients underwent surgical treatment, 22 (8.9%) patients underwent tonsillectomy, and 52 (19.8%) patients underwent revision surgery. The mean length of hospital stay was 7 days (minimum 5, maximum 11 days). Twenty-seven (10.3%) patients used antibiotics before admission.^27^ According to Gorjon, inappropriate use of prediagnosis antibiotics may cause complications.^41^ De Marie treated 8 patients with widespread parapharyngeal area infection extending to other areas, and 6 (75%) patients underwent small cervical-pharyngeal drainage with puncture drainage. However, high complication rates (4 mediastinitis, 2 pleuritis, 2 pericarditis) were observed and the mean hospitalization period was 30 days.^42^ In Broughton's study of 14 pediatric patients, 8 (57.1%) patients were treated only with i–v antibiotics. Antibiotic treatment continued for an average of 5.5 days and no complications were encountered.^43^ In our study, 13 (17.6%) patients received isolated medical treatment and 61 (82.4%) patients received medical and surgical treatment. Surgical procedures were performed under general anesthesia and no patient underwent tracheotomy during primary surgery. Two patients (2.7%) underwent tracheotomy in the postoperative period. One patient (1.4%) with multiple abscesses underwent revision surgery due to deterioration in general condition. Ampicillin sulbactam plus clindamycin combination was the most common choice for empiric antibiotic treatment (66.2%). Empirical antibiotic treatment was changed in 7 (9.4%) patients. Fifty-six (75.6%) patients had a history of antibiotic use before admission. The mean time from the onset of the complaints until admission to hospital was 7.32 days (minimum 0, maximum 47 days). The patients continued antibiotic treatment for a further 10 days after discharge. The mean duration of antibiotic use was 17.99 days (minimum 12, maximum 31 days). The mean hospitalization time was 8.28 days (minimum 2, maximum 22 days). Hospitalized patients underwent surgery on average 1.8 days (minimum 1, maximum 13 days) after admission. There was no statistically significant difference between the groups with parapharyngeal abscess and other groups in terms of hospitalization time (*p* = 0.316). Surgery was performed in 23 (76.6%) of 30 patients with isolated peritonsillar abscess and in 2 (100%) of 2 patients with peritonsillar plus parapharyngeal abscess. Abscess drainage was sufficient in 15 (60%) patients with peritonsillar abscess. 10 (40%) patients had to undergo tonsillectomy for abscess drainage.

In the Cordesmeyer study, pathology material was obtained from 49 (77.7%) patients, and malignancy was detected in 1 (2%) of these patients.^2^ Wang emphasized the importance of biopsy because pharyngeal carcinomas may present as a consequence of deep neck infection exploration.^39^ In Lin's study, malignancy was found in 4.9%, while Ridder's rate was 12.5%.^44^, ^45^ In our study, pathological material was obtained from 36 (48.6%) patients. Malignancy was found in 1 (2.7%) of the patients (cervical esophageal squamous cell carcinoma).

In the Cordesmeyer study, 60 (95.2%) patients found bacterial growth as a result of bacterial culture. In 43 (71.7%) of 60 patients with culture growth, a single bacterium grew. Mixed growth (multiple pathogens and aerobic plus anaerobic bacteria) was detected in 17 (28.3%) patients. In total, 22 different bacteria were produced and the most common microorganism (26.7%) was *Streptococcus viridans*. Among anaerobic bacteria, the most common Bacteroides (8.3%) species were grown.^2^ *Streptococcus pyogenes* was the most commonly produced microorganism in Celakovsky's study, while coagulase negative staphylococci were the most commonly produced microorganisms in Rizzo's study.^31^, ^46^ In the Shimizu study, it was noted that anaerobic bacterial growth was higher in adults (*p* = 0.02). In addition, *staphylococcus* growth was found to be significantly higher in the pediatric age group (*p* < 0.0001). However, there was no such difference in terms of streptococcal growth^25^ (*p* = 0.05). In our study, culture was taken from 60 (81.1%) patients. Bacterial growth occurred in 51 (85%) patients. Twenty-nine (48.3%) patients had isolated aerobic bacteria, 10 (16.7%) patients isolated anaerobic bacteria, 12 (20%) patients aerobic plus anaerobic bacteria (mixed, polymicrobial) growth. Among the aerobic bacteria, the most common growth rate was Streptococcus spp. (alpha hemolytic streptococci) with 34.7%. The most common anaerobic bacteria were *Peptostreptococcus spp*., *Prevotella spp*., Prevotella oralis (18.5%). *Klebsiella pneumoniae* was detected in 2 (2.7%) patients, *Brucella melitensis* in 1 (1.4%) patient and *Mycobacterium tuberculosis* bacteria in 1 (1.4%) patient.

Çetin tried isolated medical treatment in 12 pediatric patients; no complications and death were observed (101). In the Ban study, tracheotomy was performed in 32 (32.9%) patients.^28^ Crespo stated that the rate of tracheotomy was 9.2%, the complication rate was 26.1% and the mortality rate was 7.6% (102). Complications were seen in 3 (4%) patients in our study. 1 (1.4%) patient had upper airway obstruction, 1 (1.4%) patient had preterm birth, 1 (1.4%) patient had mediastinitis plus pleural effusion plus upper airway obstruction + septic shock + multiorgan failure. Two (2.7%) patients were followed in the intensive care unit, and 2 (2.7%) patients required elective tracheotomy. Our mortality rate was 1.4%. The only patient who died had additional comorbidities, a long hospital stay, and poor self-care.

## Conclusions

DNI is a disease group that seriously concerns public health with mortality and morbidity. Ensuring airway safety of patients should be the first intervention. If there is no contraindication, it is necessary to confirm the diagnosis with CT. Hospitalized patients should be examined in a multidisciplinary manner. Intubation without tracheotomy may depend on the experience of the anesthesiologist and the presence of appropriate equipment. Abscesses located lateral to the tonsil capsule may not properly drain without concomitant tonsillectomy.

## Funding

This thesis was supported by Mersin University Scientific Research Projects Unit with the number 2016-2-TP3-1825 (Mersin, Turkey).

## Conflicts of interest

The authors declare no conflicts of interest.
